# The Kids’ Environment and Health Cohort: a novel administrative data resource for research on the environmental determinants of child health in England

**DOI:** 10.23889/ijpds.v9i5.2754

**Published:** 2024-09-18

**Authors:** Selin Akaraci, Alison Macfarlane, Amal Rammah, Emily Courtin, Faith Miller, Jessica Mitchell, Joana Cruz, Matthew Lilliman, Niloofar Shoari, Samantha Hajna, Steven Cummins, Vahe Nafilyan, Pia Hardelid

**Affiliations:** 1 University College London; 2 City University of London; 3 The London School of Economics and Political Science; 4 Brock University; 5 London School of Hygiene and Tropical Medicine; 6 Office for National Statistics

## Objective and Approach

The environment in and around children’s homes and schools can influence their health and educational outcomes. Better understanding of how these potentially modifiable environmental risk factors can affect children is crucial in enabling the creation of healthier and more equitable places. We aim to establish the Kids’ Environment and Health Cohort, a research-ready, de-identified, national longitudinal birth cohort of approximately 11 million children born in England from 2006 to 2023, updated annually. The cohort will link vital statistics, census, health, education, and environmental data, via unique property identifiers from longitudinal health service address records for children and their mothers during pregnancy. Data on environmental exposures around schools will be linked to the cohort via education records. The cohort will be held and accessed in a secure research environment at the Office for National Statistics (ONS). All geographical identifiers will be encrypted and stored separately from the main cohort by the ONS to ensure privacy and security.

## Results

We have received ethics approval and have agreed the legal bases for establishing the cohort. We are now setting up data sharing agreements with each data provider. Delivery of the cohort is scheduled for late 2025.

## Conclusion

The Kid’s Environment and Health Cohort will support policy-relevant research in exploring associations between environmental factors and children's health and educational outcomes, and assessing the effectiveness of policy interventions. It will also support interdisciplinary collaboration, guiding evidence-based decision-making for environmental, planning, and public health policies aimed at promoting children’s health and well-being.

